# Supplementary material for: CCL21 Induces Plasmacytoid Dendritic Cell Migration and Activation in a Mouse Model of Glioblastoma
Source: Cancers (Basel). 2024 Oct 12;16(20):3459. doi: 10.3390/cancers16203459 (PMC11506458; doi:10.3390/cancers16203459)
Supplement: Supplementary file 1 [file cancers-16-03459-s001.zip › cancers-3240912-supplementary.pdf]

Supplementary Material

Table S1. Reagent

Name	Company	Catalog#
IL-4	Peprotech	214-14
GM-CSF	Peprotech	315-03
Flt3-Ligand	Peprotech	250-31L
CCL21	Peprotech	250-13
OVA323-339 peptides	InvivoGen	vac-isq

Table S2. Antibody

Name	Company	Catalog#
CCL21	R&D	AF457
CIITA	Novus	NBP2-59072
CIITA	Santa Cruz	sc-376174
β-arrestin	Santa Cruz	sc-74591
CCR7	R&D	MAB3477
β-actin	Invitrogen	AM4302
LaminB1	Santa Cruz	sc-374015
Anti-rabbit IgG, HRP	Cell Signaling	7074
Anti-mouse IgG, HRP	Cell Signaling	7076
Anti-rabbit IgG, AF488	Invitrogen	A-11001
Anti-mouse IgG, AF647	Invitrogen	A-21244
CD3	Biolegend	152316
CD45	Biolegend	103108
CD11c	Biolegend	117318
CD4	Biolegend	116016
CD8	Biolegend	100753
BST2	Biolegend	127015
MHC-II	Biolegend	107648
MHC-I	eBioscience	11-5998-82
ACKR4	Invitrogen	PA5-106552
CD80	Biolegend	104739
CD86	Biolegend	105039
PD-L1	Biolegend	124315
TLR9	Biolegend	159107
IFN-a	Invitrogen	PA5-119649
IL10	Biolegend	505031
TGFb	Biolegend	141403
Foxp3	eBioscience	12-5773-82
Tbet	Biolegend	644828
RORrt	eBioscience	17-6988-80

Siglec-H	Biolegend	129603
CCR7	Biolegend	120119

Table S3. qPCR primers

Name	Forward	Reverse
GAPDH	CATCACTGCCACCCAGAAGACTG	ATGCCAGTGAGCTTCCCGTTCAG
H2-Aa	GTGTGCAGACACAACTACGAGG	CTGTCACTGAGCAGACCAGAGT
H2-Ab1	GCGACGTGGGCGAGTACC	CATTCCGGAACCAGCGCA
H2-Ea	GCTGTGGACAAAGCTAACCTGG	AGGTTCACAGGGCTTCTGGAGA
H2-Eb1	TCCAGTGGCTTTGGTCAGAGAC	CAGGTTCTCCTCCAGGTTGTAG
H2-Eb2	TGCCTCAGTAGACAGGTGCAGA	AGAGCAGACCAGGAGGTTATGG
CIITA	ACCTTCGTCAGACTGGCGTTGA	GCCATTGTATCACTCAAGGAGGC
CIITA-p1	AAGAGCTGCTCTCACGGGAAT	GGTCGGCATCACTGTTAAGGA
CIITA-p3	TCTTACCTGCCGGAGTT	GGTCGGCATCACTGTTAAGGA
CIITA-p4	GAGACTGCATGCAGGCAGCA	GGTCGGCATCACTGTTAAGGA
β-actin	CATTGCTGACAGGATGCAGAAGG	TGCTGGAAGGTGGACAGTGAGG
ACKR4	CTCACGACTACAGCCAGTACGA	ATCGCCACAACTACGGAGTTCC
IRF7	CCTCTGCTTTCTAGTGATGCCG	CGTAAACACGGTCTTGCTCCTG
MyD88	ACCTGTGTCTGGTCCATTGCCA	GCTGAGTGCAAACTTGGTCTGG

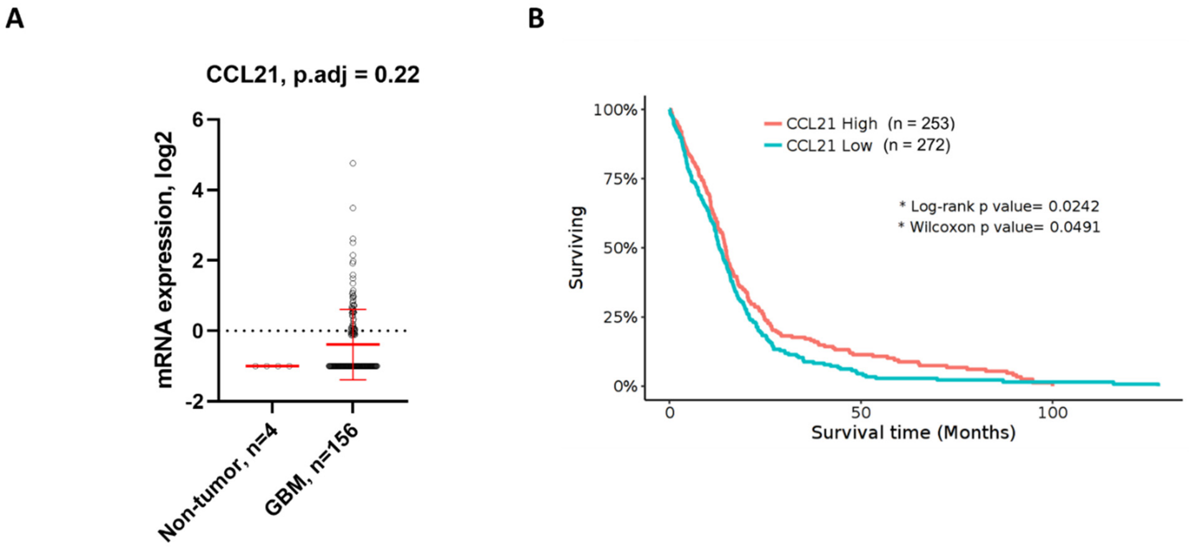

Supplemental Figure S1. Clinical relevance of CCL21 in GBM. In silico analysis of CCL21 mRNA expression and patient survival from GBM RNA-seq data in TCGA patient database.

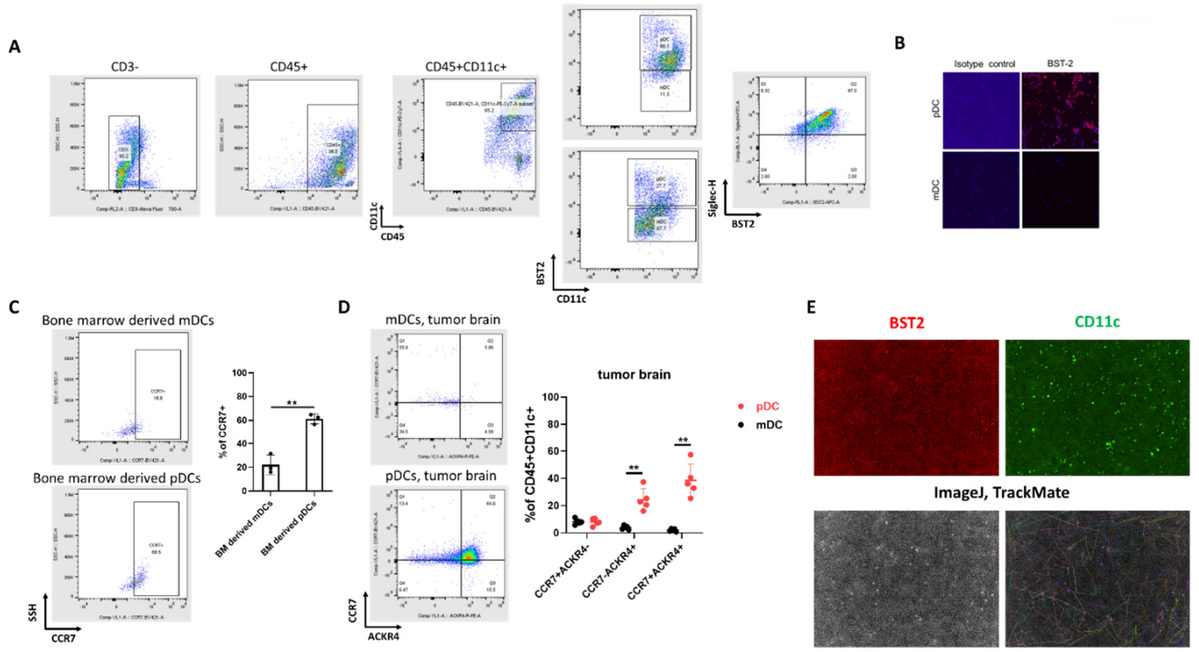

Supplemental Figure S2. Identification of pDCs and mDCs. (a) Flow cytometry gating strategy to identify pDCs and mDCs. (b) Immunocytochemistry staining of BST-2 in pDCs and mDCs. (c) CCR7 expression in Bone marrow derived pDCs and mDCs. (D) CCR7 and ACKR4

phenotype in mouse tumor bearing brain. (e) IncuCyte migration assay result was analyzed by TrackMate in ImageJ. Representative images were shown here.

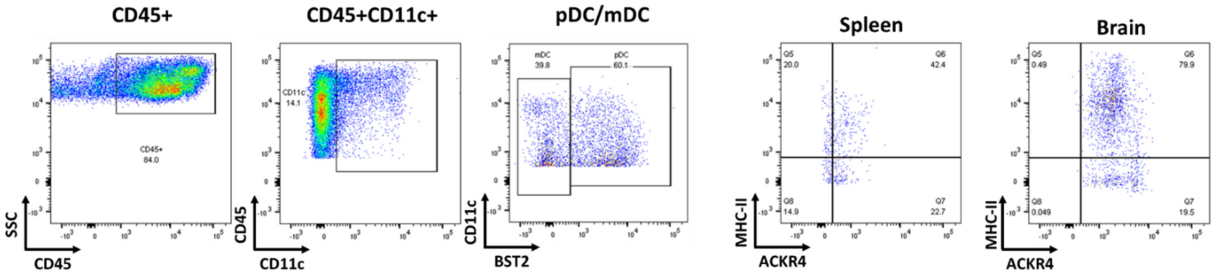

Supplemental Figure S3. ACKR4 expression in pDCs.

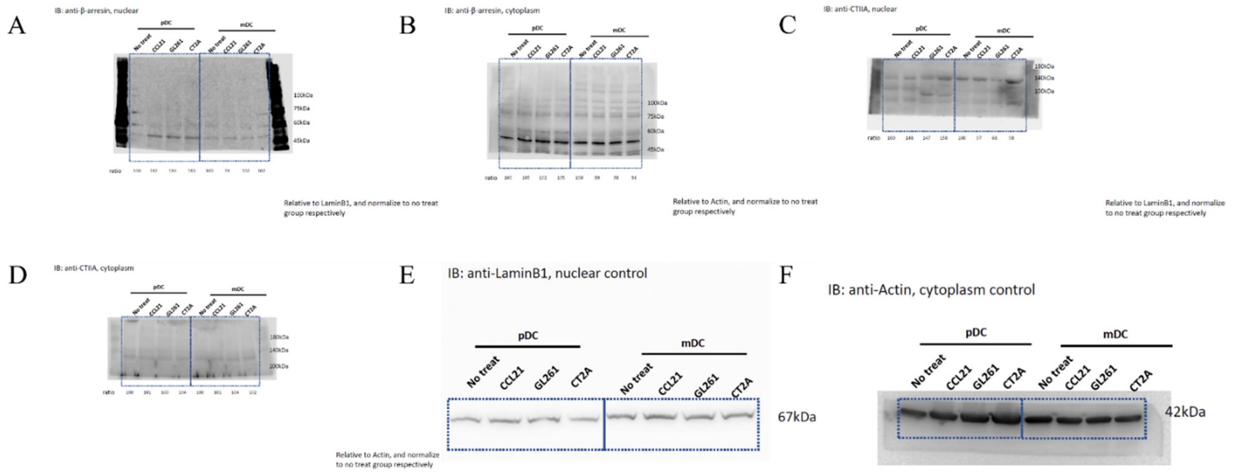

Supplemental Figure S4. Original WB images of Figure 4

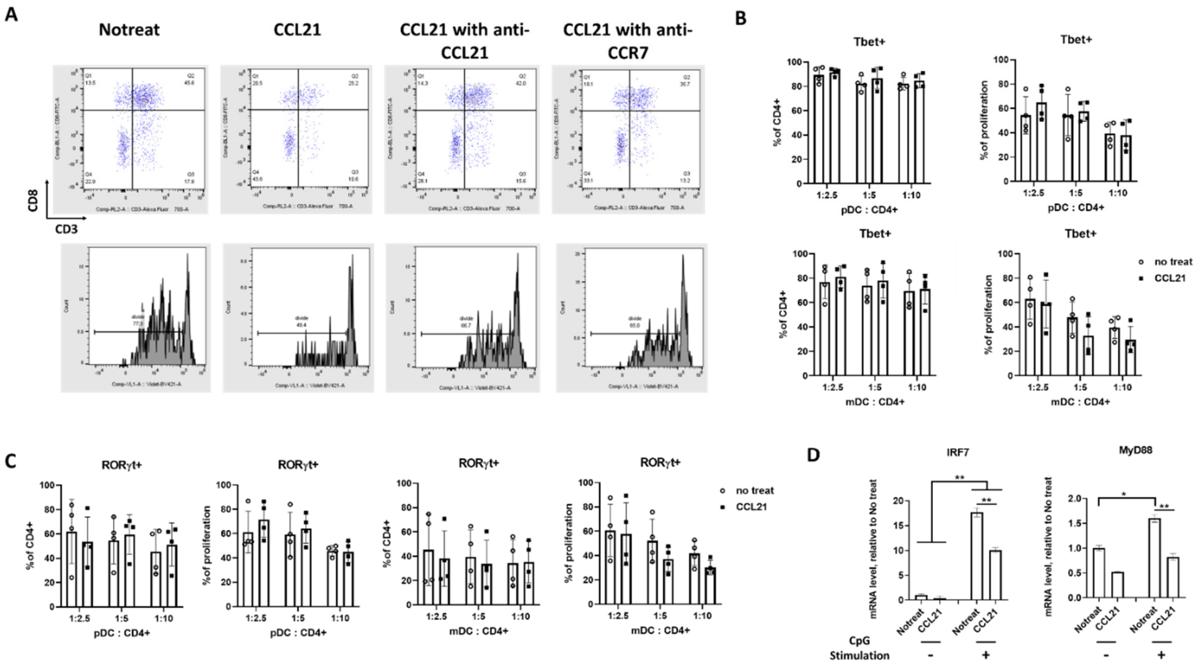

Supplemental Figure S5. CCL21 treated pDCs does not affect CD4 T cell lineage differentiation. (a) CD8+ T cells were co-culture with pretreated pDCs. T cell population and proliferation were analyzed and quantified by flow cytometry. Representative flow plots of are shown. (b) and (c) CD4+ naïve T cells were co-culture with pretreated pDCs. Th1/Tbet+ and Th2/RORγt+ T cell population and proliferation were analyzed and quantified by flow cytometry. (d) IRF7 and MyD88 gene expression in CCL21 pretreated pDCs under CpG stimulation. Data represent mean ± SEM.

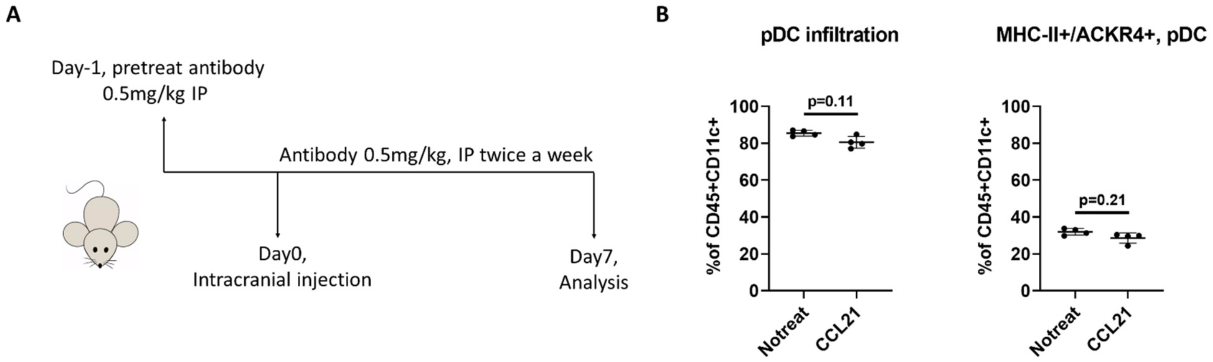

Supplemental Figure S6. Therapeutically target CCL21 in GBM mouse model. (a) CCL21 antibody administration strategy. (b) Flow cytometry analysis of pDC infiltration and activation in tumor bearing brain.
